# Learning Burnout and Internet Gaming Disorder: Longitudinal Chain Mediation Effects of Self-Control and Peer Alienation

**DOI:** 10.3390/bs15111589

**Published:** 2025-11-19

**Authors:** Xiaohui Yu, Xiaoxiao Song, Lina Li, Shaobo Lyu, Haibo Yang

**Affiliations:** 1 Faculty of Psychology, Tianjin Normal University, Tianjin 300387, China; yxh@neuq.edu.cn; 2 School of Marxism, Northeastern University at Qinhuangdao, Qinhuangdao 066004, China; 3 Hebei Key Laboratory of Mental Health and Brain Science, School of Psychology and Mental Health, North China University of Science and Technology, Tangshan 063210, China; unlex1@163.com (X.S.); lilina3725582@163.com (L.L.); lvsb@ncst.edu.cn (S.L.); 4 Institute of Developmental Psychology, Beijing Normal University, Beijing 100875, China; 5 Academy of Psychology and Behavior, Faculty of Psychology, Tianjin Normal University, Tianjin 300387, China

**Keywords:** learning burnout, internet gaming disorder, self-control, peer alienation, longitudinal mediation

## Abstract

Learning burnout, a prevalent condition among adolescents characterized by emotional exhaustion and academic disengagement, has been increasingly recognized as a critical risk factor for Internet Gaming Disorder. This study investigates the chain-mediating roles of self-control and peer alienation in this relationship. A longitudinal design was implemented with three waves of data collected from 759 Chinese middle school students. Chain mediation analyses were conducted to examine the dynamic pathways among these variables over time. Learning burnout not only directly predicted IGD but also exerted indirect effects through the mediating roles of self-control and peer alienation. The proportion of mediation effects showed an initial increase from T1 (48.18%) to T2 (60.98%), followed by a decrease at T3 (41.94%), suggesting a transition from conscious coping strategies to habitual addictive patterns. The longitudinal model (T1–T3) demonstrated that early learning burnout impaired self-control at T2, which subsequently led to increased peer alienation at T3, ultimately contributing to IGD, with this pathway accounting for 72.14% of the total effect. These findings support a dual-process mechanism involving resource depletion and compensatory need satisfaction, highlighting the importance of early interventions focused on enhancing self-regulation capabilities and improving peer relationships to prevent IGD development among adolescents.

## 1. Introduction

With the rapid development of the internet, particularly the widespread use of mobile internet, the issue of adolescents’ addiction to online gaming has become a global public health concern. In 2018, the World Health Organization (WHO) included “Gaming Disorder” in the International Classification of Diseases (ICD-11), further emphasizing its significance in public health (World Health Organization, 2018). Internet gaming disorder refers to individuals’ inability to control their use of online gaming, resulting in prolonged immersion that disrupts daily life, social interactions, and academic performance (Chi et al., 2020; Y. Pan et al., 2020). Research indicates that excessive online gaming is closely linked to learning burnout and mental health risks (Wong et al., 2020; González-Bueso et al., 2018). Learning burnout is characterized by emotional, cognitive, and behavioral exhaustion resulting from prolonged academic pressure, a lack of achievement, and a gradual decline in interest in academic pursuits (Schaufeli et al., 2020). A longitudinal study involving 293 elementary school students found that early learning burnout positively predicts later internet gaming disorder (Wang, 2022).

As a negative psychological state resulting from the accumulation of long-term academic pressure, learning burnout often accompanies a decline in self-control (Inzlicht et al., 2021) and peer alienation (Aliri et al., 2019). Based on the Conservation of Resources (COR) theory, individuals strive to acquire, protect, and maintain limited resources (such as time, energy, and social support). When these resources are threatened or lost, stress arises, leading to maladaptive behaviors (Hobfoll, 1989). Self-control refers to the conscious regulation of one’s cognition, emotions, and behaviors, and is negatively correlated with the risk of excessive internet use (Song & Park, 2019). Therefore, this study posits that learning burnout can be viewed as a state of chronic stress that may lead to the depletion of self-control resources, subsequently prompting gaming behaviors. Drawing from Self-Determination Theory (SDT), human behavior is driven by three fundamental psychological needs—autonomy, competence, and relatedness. Peer relationships are one of the most prominent interpersonal connections during adolescence (Li, 2023). Research indicates that peer relationships play a critical role in the development of children and adolescents, significantly impacting their academic, social, and emotional adaptation. Learning burnout may weaken students’ sense of competence (decreasing academic efficacy) and relatedness (peer alienation), prompting them to seek virtual achievement and social connection through gaming to fulfill unmet needs (Ryan et al., 2016). Finally, self-control significantly predicts peer alienation, with individuals possessing strong self-control being better at regulating impulsive behaviors (Duckworth et al., 2016) and promoting cooperation, thereby exhibiting higher adaptability in social interactions.

In summary, extensive research has provided evidence for the mechanism through which learning burnout influences Internet Gaming Disorder (IGD): learning burnout not only directly and positively predicts IGD but also adversely affects individuals’ self-regulatory systems by depleting self-control resources (Baumeister et al., 2007; B. Pan et al., 2016). Lower levels of self-control have been identified as a significant risk factor for externalizing problem behaviors, such as IGD (Denham et al., 2012). At the social level, learning burnout can directly and negatively predict the quality of peer relationships, and poor peer relationships themselves contribute to increased adolescent problem behaviors (J. P. Allen et al., 2006; Santor et al., 2000; Sullivan, 2006). Additionally, diminished self-control resulting from burnout can predict peer rejection in adolescents, further triggering more behavioral issues (A. Zhang, 2021). However, most existing studies have focused on cross-sectional analyses at a single time point, neglecting the dynamic mediating roles of self-regulation and social relationships. Few studies have incorporated both self-control and peer relationships into a unified model for examination.

**Figure 1:**
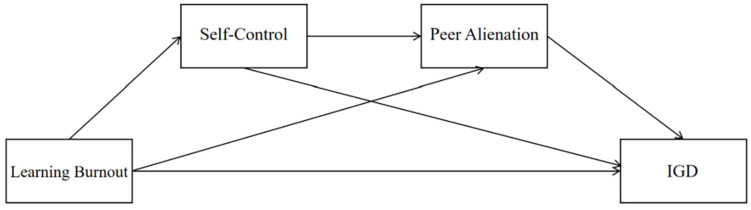
Theoretical model diagram.

To address these gaps, this study integrates Conservation of Resources (COR) Theory and Self-Determination Theory (SDT) to propose a dual-pathway model of “resource depletion–need compensation,” as illustrated in Figure 1. This model hypothesizes that under academic pressure, adolescents may either passively fall into gaming addiction due to depleted self-control resources (resource depletion pathway) or turn to the gaming world to compensate for a lack of belonging caused by peer alienation (need compensation pathway). More importantly, self-control and peer alienation are expected to form a serial mediation pathway, “learning burnout → decreased self-control → peer alienation → IGD,” reflecting the sequential process whereby psychological resource depletion leads to the deterioration of social relationships.

Based on the above theoretical and empirical foundations, and to systematically test the “resource depletion–need compensation” dual-pathway model while uncovering the dynamic mechanisms among variables, this study proposes the following specific hypotheses:

**Hypothesis 1 (H1).** 
*Self-control plays a significant mediating role in the relationship between learning burnout and IGD*.

**Hypothesis 2 (H2).** 
*Peer alienation plays a significant mediating role in the relationship between learning burnout and IGD*.

**Hypothesis 3 (H3).** 
*Self-control and peer alienation play a significant serial mediating role in the relationship between learning burnout and IGD, and this serial mediation model remains stable across different time points (T1, T2, T3)*.

## 2. Methods

### 2.1. Participants

This study adopted a convenience sampling method, selecting multiple public junior high schools from a province in Northern China. After obtaining support from the school authorities, the research team conducted the survey. Questionnaires were distributed and collected on-site within a unified time frame through homeroom teachers and psychological counselors in each class. A three-wave longitudinal design was employed, with each wave spaced four months apart. At the T1 time point, each student was assigned a unique anonymous identification code to protect privacy while enabling data matching. Under guidance, students independently completed paper-based questionnaires, resulting in 2018 valid samples as baseline data. At the T2 and T3 time points, follow-up questionnaires were administered in the same classes, with data matched using the anonymous identification codes. A total of 759 students provided valid responses across all three waves, with a mean age of 13.73 years (SD = 0.72). The sample included 353 boys and 406 girls.

### 2.2. Research Instruments

#### 2.2.1. Adolescent Learning Burnout Scale

The Adolescent Learning Burnout Scale developed by Wu et al. (2010) was used to measure learning burnout among middle school students. This scale consists of 16 items, rated on a 5-point Likert scale from “strongly agree” to “strongly disagree,” with some reverse-scoring items. Higher scores indicate higher levels of learning burnout. The scale comprises three dimensions and example items include: “Recently I’ve been feeling emotionally empty and unsure of what to do” (emotional exhaustion), “My academic performance is so poor that I really want to give up” (academic alienation) and “I am able to devote myself to studying with full energy” (low achievement). The Cronbach’s alpha coefficients of the scale at three time points were 0.87 (T1), 0.80 (T2), and 0.81 (T3).

#### 2.2.2. Internet Gaming Disorder Scale (IGD-20)

The internet gaming disorder scale, based on the DSM-V diagnostic criteria, was developed by Pontes et al. (2014) and revised by Qin et al. (2020) for the Chinese version. This scale contains 20 items, rated on a 5-point Likert scale from “strongly disagree” to “strongly agree,” with some reverse-scoring items. Higher scores indicate a greater level of internet gaming disorder. An individual is classified as having internet gaming disorder if their score exceeds 71 on this scale. The scale includes six dimensions and example items include “I often sacrifice sleep due to prolonged online gaming” (salience), “I never play online games just to make myself feel better” (emotion regulation), “Over the past year, the amount of time I spend playing online games has increased significantly” (tolerance), “I feel upset when I am unable to play online games” (withdrawal), “Because of playing online games, I have lost interest in other hobbies” (conflict), and “I want to reduce the time I spend on online games, but find it very difficult” (relapse). Example items include: “I feel restless when I cannot play online games” (withdrawal) and “I continue gaming despite knowing it causes problems” (conflict). The Cronbach’s alpha coefficients at three time points were 0.89 (T1), 0.92 (T2), and 0.95 (T3).

#### 2.2.3. Self-Control Scale (SCS)

The Self-Control Scale was developed by Tangney and revised by Tan and Guo (2008). This scale contains 19 items, rated on a 5-point Likert scale from “does not apply at all” to “applies completely,” with some reverse-scoring items. Higher scores indicate stronger self-control abilities. The scale consists of five dimensions and example items include: “People say I am impulsive” (impulse control), “It is difficult for me to break bad habits” (healthy habits), “Sometimes I get distracted by fun activities and fail to complete tasks on time” (focused work), “I am good at resisting temptations” (resisting temptation), and “I sometimes do things that bring me pleasure but are harmful to myself” (moderation in entertainment). The Cronbach’s alpha coefficients at three time points were 0.89 (T1), 0.81 (T2), and 0.89 (T3).

#### 2.2.4. Peer Alienation Scale

The Peer Alienation Scale was adapted from the Inventory of Parent and Peer Attachment (IPPA) developed by Armsden and Greenberg (1987) and revised into Chinese by Y. L. Zhang et al. (2011). This subscale consists of 7 items (“Talking over my problems with my friends makes me feel ashamed or foolish”, “I feel the need to be in touch with my friends more often”, “My friends don’t understand what I’m going through these days”, “I feel alone or apart when I am with my friends”, “I feel angry with my friends”, “I get upset a lot more than my friends know about” and “It seems as if my friends are irritated with me for no reason”.), rated on a 5-point scale, with higher scores indicating greater peer alienation. The Cronbach’s alpha coefficients at three time points were 0.86 (T1), 0.89 (T2), and 0.88 (T3).

### 2.3. Data Processing and Analysis

Data analysis was performed using SPSS 25.0 for descriptive statistics and correlation analyses. To examine the mediating roles of self-control and peer alienation in the relationship between learning burnout and Internet Gaming Disorder (IGD), we conducted serial mediation model tests using the PROCESS macro (Version 4.1, Model 6) for SPSS (Hayes, 2018). The analysis followed a logical structure to investigate both concurrent and delayed effects:

First, we examined the mechanisms of the concurrent effects of learning burnout on IGD at the same time point. Separate concurrent serial mediation models were constructed for T1, T2, and T3, with learning burnout at each time point as the independent variable, IGD as the dependent variable, and self-control and peer alienation as mediators. The testing of each model involved the following steps:

(1) Testing the predictive effect of learning burnout on self-control.
(2) Testing the predictive effect of self-control on peer alienation, while controlling for learning burnout.
(3) Testing the predictive effects of learning burnout, self-control, and peer alienation on IGD when included together in the regression model.
(4) Using the Bootstrap method to verify the significance of the three mediation paths: “learning burnout → self-control → IGD,” “learning burnout → peer alienation → IGD,” and “learning burnout → self-control → peer alienation → IGD.”

Subsequently, to explore the longitudinal predictive relationships between variables across time, a longitudinal serial mediation model from T1 to T3 was constructed. This model used T1 learning burnout as the independent variable, T3 IGD as the dependent variable, and T2 self-control and T3 peer alienation as mediators. The analytical steps were consistent with the concurrent models, aiming to examine the significance of the three longitudinal paths: “T1 learning burnout → T2 self-control → T3 IGD,” “T1 learning burnout → T3 peer alienation → T3 IGD,” and “T1 learning burnout → T2 self-control → T3 peer alienation → T3 IGD.”

## 3. Results

### 3.1. Correlation Analysis of Main Variables

Pearson correlation analysis was conducted to examine the relationships among the four variables across the three time points (T1, T2, and T3). The results indicated that all variables were pairwise correlated at T1, T2, and T3, as shown in Table 1. It should be noted that, to enhance the readability of the table and focus on the dynamic relationships among variables, Table 1 omits the autocorrelations of the same variable across different time points (e.g., the correlations between T1 learning burnout and T2, T3 learning burnout), as these correlations are typically high and anticipated.

### 3.2. Chain Mediation Model Examination of Learning Burnout and Internet Gaming Disorder

In this section, learning burnout is treated as the independent variable (X), self-control as the mediator variable 1 (M1), peer alienation as the mediator variable 2 (M2), and internet gaming disorder as the dependent variable (Y). The chain mediation effect was examined using Model 6 from the PROCESS macro version 4.1 in SPSS. The non-parametric percentile bootstrap method was applied, with 5000 resamples to estimate the 95% confidence interval (CI); if the 95% CI does not include 0, it indicates a significant mediation effect.

#### 3.2.1. T1 Period

**Figure 2:**
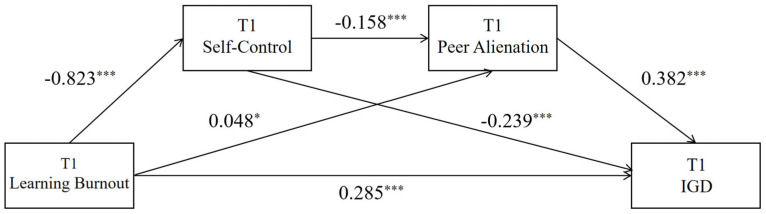
Chain Mediation Model of Self-Control and Peer Alienation Between Learning Burnout and Internet Gaming Disorder at T1. Note: * *p* < 0.05; *** *p* < 0.001.

The results showed that T1 academic burnout significantly predicted T1 self-control (β = −0.823, *p* < 0.001) and T1 peer alienation (β = 0.048, *p* < 0.05). T1 self-control significantly predicted T1 peer alienation (β = −0.158, *p* < 0.001). Additionally, T1 academic burnout, T1 self-control, and T1 peer alienation all significantly predicted T1 problematic online gaming use (β = 0.285, −0.239, and 0.382, respectively; all *p* < 0.001). See Table 2 and Figure 2 for details.

From the model perspective, T1 learning burnout impacts internet gaming disorder through four pathways: the direct path (direct): T1 learning burnout → T1 internet gaming disorder; indirect path 1 (indirect1): T1 Learning Burnout → T1 self-control → T1 internet gaming disorder; indirect path 2 (indirect2): T1 Learning Burnout → T1 peer alienation → T1 internet gaming disorder; indirect path 3 (indirect3): T1 Learning Burnout → T1 self-control → T1 peer alienation → T1 internet gaming disorder. According to the data analysis results in Table 3, the direct effect of the chain mediation model is 0.285, and the 95% CI does not include 0, indicating a significant direct effect; the total mediation effect is 0.265, accounting for 48.18% of the total effect.

#### 3.2.2. T2 Period

**Figure 3:**
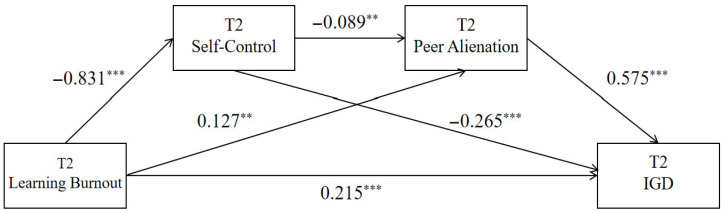
Chain Mediation Model of Self-Control and Peer Alienation Between Learning Burnout and Internet Gaming Disorder at T2. Note: ** *p* < 0.01; *** *p* < 0.001.

The results showed that T2 academic burnout significantly predicted T2 self-control (β = −0.831, *p* < 0.001) and T2 peer alienation (β = 0.127, *p* < 0.001). T2 self-control significantly predicted T2 peer alienation (β = −0.249, *p* < 0.001). Additionally, T2 academic burnout, T2 self-control, and T2 peer alienation all significantly predicted T2 problematic online gaming use (β = 0.215, −0.265, and 0.575, respectively; all *p* < 0.001). See Table 4 and Figure 3 for details.

From the model perspective, T2 learning burnout impacts internet gaming disorder through four pathways: the direct path (direct): T2 learning burnout → T2 internet gaming disorder; indirect path 1 (indirect1): T2 learning burnout → T2 self-control → T2 internet gaming disorder; indirect path 2 (indirect2): T2 learning burnout → T2 peer alienation → T2 internet gaming disorder; indirect path 3 (indirect3): T2 learning burnout → T2 self-control → T2 peer alienation → T2 internet gaming disorder. According to the data analysis results presented in Table 5, the direct effect of the chain mediation model is 0.215, and the 95% confidence interval (CI) does not include 0, indicating a significant direct effect. The total mediation effect is 0.336, accounting for 60.98% of the total effect.

#### 3.2.3. T3 Period

**Figure 4:**
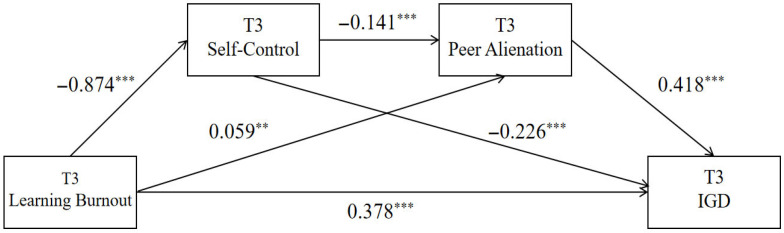
Chain Mediation Model of Self-Control and Peer Alienation Between Learning Burnout and Internet Gaming Disorder at T3. Note: ** *p* < 0.01; *** *p* < 0.001.

The results (Table 6 and Figure 4) indicate that T3 academic burnout significantly predicted T3 self-control (β = −0.874, *p* < 0.001) and T3 peer alienation (β = 0.059, *p* < 0.01). T3 self-control significantly predicted T3 peer alienation (β = −0.141, *p* < 0.001). Furthermore, T3 academic burnout, T3 self-control, and T3 peer alienation all positively predicted T3 problematic online gaming use (β = 0.378, −0.226, and 0.418, respectively; all *p* < 0.001).

From the model perspective, T3 Learning Burnout impacts internet gaming disorder through four pathways: the direct path (direct): T3 Learning Burnout → T3 internet gaming disorder; indirect path 1 (indirect1): T3 Learning Burnout → T3 self-control → T3 internet gaming disorder; indirect path 2 (indirect2): T3 Learning Burnout → T3 peer alienation → T3 internet gaming disorder; and indirect path 3 (indirect3): T3 Learning Burnout → T3 self-control → T3 peer alienation → T3 internet gaming disorder. According to the data analysis results presented in Table 7, the direct effect of the chain mediation model is 0.378, and the 95% confidence interval (CI) does not include 0, indicating a significant direct effect. The total mediation effect is 0.273, accounting for 41.94% of the total effect.

#### 3.2.4. Longitudinal Mediation from T1 to T3

**Figure 5:**
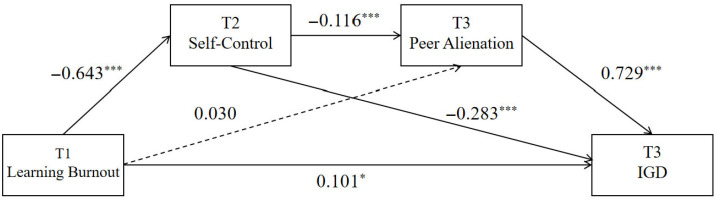
Chain Mediation Model of Self-Control and Peer Alienation between Learning Burnout and Internet Gaming Disorder from T1 to T3. Note: * *p* < 0.05; *** *p* < 0.001; The dashed line represents a path that is not significant.

The results (Table 8 and Figure 5) show that T1 academic burnout significantly predicted T2 self-control (β = −0.643, *p* < 0.001), but its predictive effects on T3 peer alienation were not significant (β = 0.030, *p* > 0.05). T2 self-control significantly predicted T3 peer alienation (β = −0.116, *p* < 0.001). Additionally, T1 academic burnout, T2 self-control, and T3 peer alienation all significantly predicted T3 problematic online gaming use (β = 0.101, *p* < 0.05; β = −0.283 and 0.729, *p* < 0.001, respectively).

From the model perspective, T1 learning burnout impacts internet gaming disorder through three pathways: the direct path (direct): T1 learning burnout → T3 internet gaming disorder; indirect path 1 (indirect1): T1 learning burnout → T2 self-control → T3 internet gaming disorder; and indirect path 3 (indirect3): T1 learning burnout → T2 self-control → T3 peer alienation → T3 internet gaming disorder. Analysis results from Table 9 indicate that the direct effect of this chain mediation model was 0.100, with a 95% confidence interval excluding 0, suggesting a significant direct effect. The total indirect effect was 0.359, accounting for 72.14% of the total effect.

## 4. Discussion

This study employed a three-wave longitudinal design to systematically investigate the mechanisms through which learning burnout influences Internet Gaming Disorder (IGD). First, while the direct predictive path from academic pressure alone to IGD was significant, it was notably weak. Nevertheless, its significance indicates an independent long-term effect. Second, the majority of the model’s influence (72.14%) was transmitted through the two mediating variables of “self-control” and “peer alienation.” This suggests that, in the long term, learning burnout leads to gaming addiction by depleting students’ psychological resources (self-control) and damaging their social relationships (peer alienation). The results provide a detailed analysis of the independent and serial mediating roles of self-control and peer alienation, revealing the dynamic characteristics of this mechanism over time.

### 4.1. The Mediating Role of Self-Control

The findings support Hypothesis 1, confirming that self-control plays a key mediating role between learning burnout and IGD. This strongly aligns with the Conservation of Resources (COR) theory. Learning burnout can be viewed as a chronic state of resource depletion resulting from prolonged academic pressure. In this state, the psychological resources available for self-regulation are continuously depleted, leading to a decline in self-control. As demonstrated in this study, learning burnout at all three time points significantly and negatively predicted self-control levels. Individuals under high pressure, due to resource depletion, fall into a state of dyscontrol (Gentile et al., 2017; Bender et al., 2020), making it difficult for adolescents to resist the immediate allure of online games and manage their gaming time effectively, thereby significantly increasing the risk of IGD. This “resource depletion pathway” illustrates the internal process through which individuals, under academic pressure, transition from active cognitive engagement to passive behavioral dyscontrol, ultimately leading to gaming addiction. Our longitudinal model (T1 → T3) further underscores the central role of self-control. The path from T1 learning burnout to T3 IGD via T2 self-control had the largest effect size among all mediating paths, highlighting the importance of replenishing and enhancing adolescents’ self-control resources in early interventions (Duckworth et al., 2019).

### 4.2. The Mediating Role of Peer Alienation

This study also validated Hypothesis 2, indicating that peer alienation is another significant pathway through which learning burnout affects IGD. This path can be interpreted through the lens of Self-Determination Theory. Learning burnout not only depletes cognitive resources but also undermines adolescents’ sense of competence and relatedness. When students experience frustration and alienation in the academic environment, their emotional connections with peers may weaken, leading to peer alienation (Aliri et al., 2019). Consequently, they may turn to the online gaming world, where immediate feedback, clear achievements, and a sense of group belonging are more readily available. Thus, the peer alienation mediation pathway reveals a “need compensation pathway”: academic pressure leads to unfulfilled social needs in reality, which in turn drives individuals to seek substitute satisfaction in virtual spaces, ultimately resulting in excessive reliance on games (Kowert et al., 2013). Although the independent mediating role of peer alienation was not significant in the longitudinal model (T1 → T3), its stable role in the concurrent models suggests it remains a risk factor that cannot be overlooked (Marino et al., 2018).

### 4.3. The Serial Mediating Role of Self-Control and Peer Alienation

The core finding of this study supports Hypothesis 3, demonstrating that self-control and peer alienation form a longitudinal serial mediation chain between learning burnout and IGD. This reveals a dynamic, sequential developmental process: early learning burnout first depletes an individual’s self-control resources (resource depletion); subsequently, impaired self-control makes individuals more prone to impulsive, negative, or withdrawn behaviors in interpersonal interactions (Duckworth et al., 2016), hindering the maintenance of high-quality peer relationships and thereby exacerbating peer alienation; finally, the combination of low self-control and social isolation in reality pushes individuals toward online games as an outlet for venting negative emotions and compensating for social needs, significantly increasing the risk of IGD (Brand et al., 2019).

Furthermore, the mediating effects across the T1–T3 periods showed an initial increase (T1: 48.18% → T2: 60.98%) followed by a decrease (T3: 41.94%). This pattern likely reflects the dynamic psychological process of IGD formation and intensification. During the T1–T2 period, students facing academic pressure might initially mobilize self-regulatory mechanisms to cope with challenges. However, if these regulatory efforts persistently fail, they can accelerate resource depletion. Over time, as self-control resources are further exhausted, individuals become less capable of maintaining interpersonal relationships, leading to more severe peer alienation and causing the mediating effect to peak at T2. The significant decline in the mediating effect during the T2–T3 stage aligns with the core premise of the Conservation of Resources (COR) theory (Hobfoll et al., 2018), which posits that sustained pressure leads to severe depletion of psychological resources. Prolonged resource loss may cause self-control capacity at T3 to stabilize at a low level, reducing the statistical power of its mediating role. Concurrently, peer alienation relationships may have become entrenched, diminishing their mediating efficacy. More importantly, the temporal dynamic data support a critical shift: IGD may have evolved beyond merely a “coping mechanism” for alleviating burnout, having developed addictive characteristics in its own right (Brand et al., 2016).

It should also be noted that, although this study is theory-driven and longitudinal, proposing a unidirectional path model from learning burnout through self-control and peer alienation to IGD, we fully acknowledge the potential complexity of bidirectional or reciprocal relationships between the variables. For instance, a student deeply entrenched in IGD might experience worsened academic performance, potentially exacerbating learning burnout and creating a vicious cycle. Such reverse effects likely exist and may function as mechanisms that maintain and aggravate the problem, even if they are not the initial cause. The model constructed in this study is grounded in the Conservation of Resources Theory and Self-Determination Theory, which collectively point to a clear pathway starting from “external circumstances leading to internal resource loss”—a pathway that is temporally and logically coherent and natural. The longitudinal data from this study are highly consistent with this theoretical expectation, further supporting the plausibility of learning burnout as the starting point of the mechanism. Therefore, while bidirectional influences cannot be entirely ruled out, based on the internal theoretical consistency and the empirical evidence obtained, we argue that the model positioning “learning burnout as the mechanism’s starting point” currently holds greater explanatory power. Future research could employ more inferentially powerful analytical methods, such as cross-lagged panel models, to more precisely reveal the complex bidirectional relationships between variables and quantify the relative contributions of different paths.

The findings of this study underscore the importance of staged interventions and offer new perspectives for individual difference research (Przybylski et al., 2016). During the T1–T2 phase of learning burnout, replenishing psychological resources (Tang et al., 2016) and enhancing self-control and stress-resource transformation capabilities through cognitive reappraisal-based reframing training (Gross, 2014) can effectively prevent the collapse of an individual’s self-regulatory system (Creswell, 2017). Concurrently, peer relationships can be improved through group activities to restore real-world social connections (K. A. Allen et al., 2017), such as replacing virtual gaming with collaborative learning activities to fulfill the individual’s need for belonging. By the T3 stage, however, due to resource depletion, interventions need to directly target habit disruption and addictive behaviors themselves, supplemented by addressing the root causes of learning burnout.

### 4.4. Limitations and Future Research

This study has several limitations that point to directions for future research. First, in terms of sampling, the use of a convenience sampling method resulted in a sample drawn exclusively from urban secondary schools in Northern China. This led to homogeneity in cultural, socioeconomic, and educational ecological backgrounds, thereby limiting the generalizability of the findings to rural areas and adolescent populations from other cultural contexts. Future research should adopt multi-stage cluster sampling and conduct cross-cultural comparative studies to examine the universality of the current model across diverse educational and cultural settings.

Second, it is also important to note the analytical approach adopted in this study. While we tested longitudinal mediation pathways, future research could employ autoregressive models, such as the cross-lagged panel model, to more stringently control for the stability of variables over time and provide an even more robust examination of the reciprocal relationships between learning burnout, self-control, peer alienation, and IGD.

Finally, this study did not incorporate physiological indicators, thus preventing the exploration of the physiological mechanisms underlying learning burnout and the depletion of self-control resources. Future research could integrate Ecological Momentary Assessment methods (EMA) (Stone et al., 2023) to capture the daily dynamics of academic stress, self-control fluctuations, and gaming cravings at a micro level, thereby more precisely revealing the mechanisms of problematic behavior transformation at the within-individual level.

## 5. Conclusions

This study reveals the complex mechanism through which learning burnout influences problematic online gaming, highlighting the crucial roles of self-control and peer relationships in this process. It provides a more comprehensive and dynamic picture of how these variables interact over time, gradually leading adolescents toward addiction. The findings confirm that initial academic burnout may activate the consumption of self-regulatory resources, while persistent learning burnout significantly depletes self-control capacity, thereby increasing the risk of Internet Gaming Disorder (IGD). Peer alienation further reinforces this risk, underscoring the importance of social relationships in coping with academic pressure.

This study contributes at theoretical, methodological, and practical levels. Theoretically, it innovatively integrates Conservation of Resources Theory and Self-Determination Theory to propose a dual-pathway model of “resource depletion–need compensation,” systematically elucidating the psychological process through which academic pressure leads to gaming addiction and offering an integrated perspective for understanding this issue. Methodologically, it is the first to examine and confirm the serial mediating role of self-control and peer alienation within a longitudinal framework, clarifying both the stability and dynamic evolution of this mechanism over time, thereby enhancing the credibility and depth of the findings. Practically, the results directly emphasize the importance of staged interventions: during the early stages of IGD development in middle school students, efforts should focus on replenishing self-control resources and fostering peer relationships, whereas later stages require direct habit disruption targeting addictive behaviors. This provides a critical theoretical foundation for designing precise prevention and intervention strategies. Future research could incorporate larger samples and ecological momentary assessment methods to further validate the generalizability of this model and explore its underlying physiological mechanisms.

## Figures and Tables

**Table 1:** Correlation Analysis of Variables at Different Time Points.

Var	1	2	3	4	5	6	7	8	9	10	11	12
1. T1 Learning Burnout	1											
2. T2 Learning Burnout	-	1										
3. T3 Learning Burnout	-	-	1									
4. T1 Self-Control	−0.633 ***	−0.488 ***	−0.403 ***	1								
5. T2 Self-Control	−0.468 ***	−0.671 ***	−0.545 ***	-	1							
6. T3 Self-Control	−0.359 ***	−0.484 ***	−0.678 ***	-	-	1						
7. T1 Peer Alienation	0.380 ***	0.338 ***	0.269 ***	−0.504 ***	−0.393 ***	−0.299 ***	1					
8. T2 Peer Alienation	0.312 ***	0.453 ***	0.350 ***	−0.370 ***	−0.441 ***	−0.361 ***	-	1				
9. T3 Peer Alienation	0.217 ***	0.285 ***	0.401 ***	−0.221 ***	−0.359 ***	−0.487 ***	-	-	1			
10. T1 IGD	0.485 ***	0.344 ***	0.300 ***	−0.513 ***	−0.387 ***	−0.319 **	0.392 ***	0.302 ***	0.187 **	1		
11. T2 IGD	0.339 ***	0.490 ***	0.358 ***	−0.400 ***	−0.520 ***	−0.395 ***	0.302 ***	0.442 ***	0.262 ***	-	1	
12. T3 IGD	0.272 ***	0.347 ***	0.524 ***	−0.317 ***	−0.425 ***	−0.515 ***	0.250 ***	0.285 ***	0.389 ***	-	-	1

Note: ** *p* < 0.01; *** *p* < 0.001.

**Table 2:** Chain Mediation Regression Analysis Between Learning Burnout and internet gaming disorder at T1.

Dependent Variable	Predictor Variable	R	R^2^	F	β	t
T1 Self-Control	T1 Learning Burnout	0.633	0.400	505.244 ***	−0.823	−22.478 ***
T1 Peer Alienation	T1 Learning Burnout	0.510	0.260	123.82 ***	0.048	2.530 *
	T1 Self-Control				−0.158	−10.869 ***
T1 IGD	T1 Learning Burnout	0.570	0.325	121.009 ***	0.285	6.472 ***
	T1 Self-Control				−0.239	−6.613 ***
	T1 Peer Alienation				0.382	4.554 ***

Note: * *p* < 0.05; *** *p* < 0.001.

**Table 3:** Chain Mediation Effects of Self-Control and Peer Alienation Between Learning Burnout and internet gaming disorder at T1.

Effect	Path	Effect Size	SE	95% CI
Mediation Effect	T1 Learning Burnout → T1 Self-Control → T1 Internet Gaming Disorder (indirect1)	0.197	0.034	0.129–0.266
	T1 Learning Burnout → T1 Peer Alienation → T1 Internet Gaming Disorder (indirect2)	0.018	0.009	0.002–0.040
	T1 Learning Burnout → T1 Self-Control → T1 Peer Alienation → T1 Internet Gaming Disorder (indirect3)	0.050	0.013	0.026–0.077
Total Mediation Effect	indirect1 + indirect2 + indirect3	0.265	0.035	0.199–0.336
Direct Effect	T1 Learning Burnout → T1 Internet Gaming Disorder (direct)	0.285	0.044	0.198–0.371
Total Effect	Indirect Effect + Direct Effect	0.550	0.036	0.479–0.621

**Table 4:** Chain Mediation Regression Analysis Between Learning Burnout and Internet Gaming Disorder at T2.

**Dependent Variable**	**Predictor Variable**	**R**	**R^2^**	**F**	**β**	**t**
T2 Self-Control	T2 Learning Burnout	0.671	0.451	621.241 ***	−0.831	−24.925 ***
T2 Peer Alienation	T2 Learning Burnout	0.489	0.240	119.094 ***	0.127	6.687 ***
	T2 Self-Control				−0.249	−5.819 ***
T2 IGD	T2 Learning Burnout	0.588	0.346	133.048 ***	0.215	4.682 ***
	T2 Self-Control				−0.265	−7.192 ***
	T2 Peer Alienation				0.575	6.710 ***

Note: *** *p* < 0.001.

**Table 5:** Chain Mediation Effects of Self-Control and Peer Alienation Between Learning Burnout and Internet Gaming Disorder at T2.

Effect	Path	Effect Size	SE	95% CI
Mediation Effect	T2 Learning Burnout → T2 Self-Control → T2 Internet Gaming Disorder (indirect1)	0.220	0.041	0.142–0.302
	T2 Learning Burnout → T2 Peer Alienation → T2 Internet Gaming Disorder (indirect2)	0.073	0.022	0.033–0.119
	T2 Learning Burnout → T2 Self-Control → T2 Peer Alienation → T2 Internet Gaming Disorder (indirect3)	0.043	0.013	0.020–0.071
Total Mediation Effect	indirect1 + indirect2 + indirect3	0.336	0.044	0.249–0.424
Direct Effect	T2 Learning Burnout → T2 Internet Gaming Disorder (direct)	0.215	0.046	0.125–0.305
Total Effect	Indirect Effect + Direct Effect	0.551	0.036	0.481–0.620

**Table 6:** Chain Mediation Regression Analysis Between Learning Burnout and Internet Gaming Disorder at T3.

Dependent Variable	Predictor Variable	R	R^2^	F	β	t
T3 Self-Control	T3 Learning Burnout	0.678	0.459	643.269 ***	−0.874	−25.363 ***
T3 Peer Alienation	T3 Learning Burnout	0.497	0.247	123.748 ***	0.059	3.025 **
	T3 Self-Control				−0.141	−9.303 ***
T3 IGD	T3 Learning Burnout	0.583	0.340	129.466 ***	0.378	7.521 ***
	T3 Self-Control				−0.226	−5.515 ***
	T3 Peer Alienation				0.418	4.490 ***

Note: ** *p* < 0.01; *** *p* < 0.001.

**Table 7:** Chain Mediation Effects of Self-Control and Peer Alienation Between Learning Burnout and Internet Gaming Disorder at T3.

Effect	Path	Effect Size	SE	95% CI
Mediation Effect	T3 Learning Burnout → T3 Self-Control → T3 Internet Gaming Disorder (indirect1)	0.197	0.039	0.012–0.276
	T3 Learning Burnout → T3 Peer Alienation → T3 Internet Gaming Disorder (indirect2)	0.025	0.012	0.006–0.051
	T3 Learning Burnout → T3Self-Control → T3 Peer Alienation → T3 Internet Gaming Disorder (indirect3)	0.051	0.017	0.022–0.087
Total Mediation Effect	indirect1 + indirect2 + indirect3	0.273	0.039	0.197–0.354
Direct Effect	T3 Learning Burnout → T3 Internet Gaming Disorder (direct)	0.378	0.050	0.280–0.477
Total Effect	Indirect Effect + Direct Effect	0.651	0.039	0.576–0.727

**Table 8:** Longitudinal Mediation Analysis from T1 to T3.

Dependent Variable	Predictor Variable	R	R^2^	F	β	t
T2 Self-Control	T1 Learning Burnout	0.468	0.219	212.794 ***	−0.643	−14.587 ***
T3 Peer Alienation	T1Learning Burnout	0.363	0.132	57.424 ***	0.030	1.635
	T2 Self-Control				−0.116	−8.592 ***
T3 IGD	T1 Learning Burnout	0.500	0.250	83.695 ***	0.101	2.128 *
	T2 Self-Control				−0.283	−7.861 ***
	T3 Peer Alienation				0.729	7.891 ***

Note: * *p* < 0.05; *** *p* < 0.001.

**Table 9:** Longitudinal Chain Mediation Effects of Self-Control and Peer Alienation between Learning Burnout and Internet Gaming Disorder from T1 to T3.

Effect	Path	Effect Size	SE	95% CI
Mediation Effect	T1 Learning Burnout → T2 Self-Control → T3 internet gaming disorder (indirect1)	0.182	0.028	0.129–0.236
	T1 Learning Burnout → T3 Peer Alienation → T3 internet gaming disorder (indirect2)	0.022	0.014	−0.005–0.052
	T1 Learning Burnout → T2 Self-Control → T3 Peer Alienation → T3 internet gaming disorder (indirect3)	0.055	0.012	0.033–0.082
Total Mediation Effect	indirect1 + indirect2 + indirect3	0.259	0.031	0.200–0.320
Direct Effect	T1 Learning Burnout → T3 internet gaming disorder (direct)	0.100	0.047	0.008–0.193
Total Effect	Indirect Effect + Direct Effect	0.359	0.046	0.268–0.450

## Data Availability

The data presented in this study are available upon request from the corresponding author. The data are not publicly available due to participant confidentiality and institutional ethical guidelines.

## Abbreviations

The following abbreviations are used in this manuscript:

WHO	World Health Organization
ICD	International Classification of Diseases
COR	Conservation of Resources
SDT	Self-Determination Theory
IGD	Internet Gaming Disorder
SCS	Self-Control Scale

## References

[B1-behavsci-15-01589] Aliri J., Muela A., Gorostiaga A., Balluerka N., Aritzeta A., Soroa G. (2019). Stressful life events and depressive symptomatology among Basque adolescents: The mediating role of attachment representations. Psychological Reports.

[B2-behavsci-15-01589] Allen J. P., Porter M. R., McFarland F. C. (2006). Leaders and followers in adolescent close friendships: Susceptibility to peer influence as a predictor of risky behavior, friendship instability, and depression. Development and Psychopathology.

[B3-behavsci-15-01589] Allen K. A., Kern M. L., Vella-Brodrick D., Waters L. (2017). School values: A comparison of academic motivation, mental health promotion, and school belonging with student achievement. Educational and Developmental Psychologist.

[B4-behavsci-15-01589] Armsden G. C., Greenberg M. T. (1987). The Inventory of parent and peer attachment: Relationships to well-being in adolescence. Journal of Youth and Adolescence.

[B5-behavsci-15-01589] Baumeister R. F., Vohs K. D., Tice D. M. (2007). The strength model of self-control. Current Directions in Psychological Science.

[B6-behavsci-15-01589] Bender P. K., Kim E. L., Gentile D. A. (2020). Gaming disorder in children and adolescents: Risk factors and preventive approaches. Current Addiction Reports.

[B7-behavsci-15-01589] Brand M., Wegmann E., Stark R., Müller A., Wölfling K., Robbins T. W., Potenza M. N. (2019). The interaction of person-affect-cognition-execution (I-PACE) model for addictive behaviors: Update, generalization to addictive behaviors beyond internet-use disorders, and specification of the process character of addictive behaviors. Neuroscience & Biobehavioral Reviews.

[B8-behavsci-15-01589] Brand M., Young K. S., Laier C., Wölfling K., Potenza M. N. (2016). Integrating psychological and neurobiological considerations regarding the development and maintenance of specific Internet-use disorders: An interaction of person-affect-cognition-execution (I-PACE) model. Neuroscience & Biobehavioral Reviews.

[B9-behavsci-15-01589] Chi X., Hong X., Chen X. (2020). Profiles and sociodemographic correlates of Internet addiction in early adolescents in southern China. Addictive Behaviors.

[B10-behavsci-15-01589] Creswell J. D. (2017). Mindfulness interventions. Annual Review of Psychology.

[B11-behavsci-15-01589] Denham S. A., Bassett H. H., Zinsser K. (2012). Early childhood teachers as socializers of young children’s emotional competence. Early Childhood Education Journal.

[B12-behavsci-15-01589] Duckworth A. L., Gendler T. S., Gross J. J. (2016). Situational strategies for self-control. Perspectives on Psychological Science.

[B13-behavsci-15-01589] Duckworth A. L., Taxer J. L., Eskreis-Winkler L., Galla B. M., Gross J. J. (2019). Self-control and academic achievement. Annual Review of Psychology 70.

[B14-behavsci-15-01589] Gentile D. A., Bailey K., Bavelier D., Brockmyer J. F., Cash H., Coyne S. M., Doan A., Grant D. S., Green C. S., Griffiths M., Markle T. (2017). Internet gaming disorder in children and adolescents. Pediatrics.

[B15-behavsci-15-01589] González-Bueso V., Santamaría J. J., Fernández D., Merino L., Montero E., Ribas J. (2018). Association between internet gaming disorder or pathological video-game use and comorbid psychopathology: A comprehensive review. International Journal of Environmental Research and Public Health.

[B16-behavsci-15-01589] Gross J. J. (2014). Emotion regulation: Conceptual and empirical foundations. Handbook of emotion regulation.

[B17-behavsci-15-01589] Hayes A. F. (2018). Introduction to mediation, moderation, and conditional process analysis: A regression-based approach.

[B18-behavsci-15-01589] Hobfoll S. E. (1989). Conservation of resources: A new attempt at conceptualizing stress. American Psychologist.

[B19-behavsci-15-01589] Hobfoll S. E., Halbesleben J., Neveu J.-P., Westman M. (2018). Conservation of resources in the organizational context: The reality of resources and their consequences. Annual Review of Organizational Psychology and Organizational Behavior.

[B20-behavsci-15-01589] Inzlicht M., Werner K. M., Briskin J. L., Roberts B. W. (2021). Integrating models of self-regulation. Annual Review of Psychology.

[B21-behavsci-15-01589] Kowert R., Domahidi E., Quandt T. (2013). (A)Social reputation: Exploring the relationship between online video game involvement and social competence. Computers in Human Behavior.

[B22-behavsci-15-01589] Li Z. H. (2023). The impact of school bullying on learning burnout in high school students: The mediating role of peer relationships and intervention research. Master’s thesis.

[B23-behavsci-15-01589] Marino C., Gini G., Vieno A., Spada M. M. (2018). The associations between problematic Facebook use, psychological distress and well-being among adolescents and young adults: A systematic review and meta-analysis. Journal of Affective Disorders.

[B24-behavsci-15-01589] Pan B., Zhang L., Zhang W., Sang B. (2016). The relationship between adolescents’ poor academic performance, learning stress, and voluntary control: A cross-lagged study. Psychological Development and Education.

[B25-behavsci-15-01589] Pan Y., Chiu Y., Lin Y. (2020). Systematic review and meta-analysis of epidemiology of internet addiction. Neuroscience & Biobehavioral Reviews.

[B26-behavsci-15-01589] Pontes H. M., Kiraly O., Demetrovics Z., Griffiths M. D. (2014). The conceptualisation and measurement of DSM-5 internet gaming disorder: The development of the IGD-20 Test. PLoS ONE.

[B27-behavsci-15-01589] Przybylski A. K., Weinstein N., Murayama K. (2016). Internet gaming disorder: Investigating the clinical relevance of a new phenomenon. Am J Psychiatry.

[B28-behavsci-15-01589] Qin L. X., Liu Q. S., Luo T. (2020). Reliability and validity of the Chinese version of the internet gaming disorder scale in college students. Chinese Journal of Clinical Psychology.

[B29-behavsci-15-01589] Ryan R. M., Deci E. L., Vansteenkiste M., Cicchetti D. (2016). Autonomy and autonomy disturbances in self-development and psychopathology: Research on motivation, attachment, and clinical process. Developmental psychopathology: Theory and method.

[B30-behavsci-15-01589] Santor D. A., Messervey D., Kusumakar V. (2000). Measuring peer pressure, popularity, and conformity in adolescent boys and girls: Predicting school performance, sexual attitudes and substance abuse. Journal of Youth and Adolescence.

[B31-behavsci-15-01589] Schaufeli W. B., Desart S., De Witte H. (2020). Burnout assessment tool (BAT)—Development, validity, and reliability. International Journal of Environmental Research and Public Health.

[B32-behavsci-15-01589] Song W. J., Park J. W. (2019). The influence of stress on internet addiction: Mediating effects of self-control and mindfulness. International *Journal of Mental Health and Addiction*.

[B33-behavsci-15-01589] Stone A. A., Schneider S., Smyth J. M. (2023). Evaluation of pressing issues in ecological momentary assessment. Annual Review of Clinical Psychology.

[B34-behavsci-15-01589] Sullivan C. J. (2006). Early adolescent delinquency assessing the role of childhood problems, family environment, and peer pressure. Youth Violence and Juvenile Justice.

[B35-behavsci-15-01589] Tan S. H., Guo Y. Y. (2008). Revision of the self-control scale for Chinese college students. Chinese Journal of Clinical Psychology.

[B36-behavsci-15-01589] Tang Y. Y., Hölzel B. K., Posner M. I. (2016). Traits and states in mindfulness meditation. Nature Reviews Neuroscience.

[B37-behavsci-15-01589] Wang Y. F. (2022). The relationship between academic burnout, avatar identification, and online gaming dependence in primary school students. Master’s thesis.

[B38-behavsci-15-01589] Wong H. Y., Mo H. Y., Potenza M. N., Chan M. N. M., Lau W. M., Chui T. K., Pakpour A. H., Lin C. Y. (2020). Relationships between severity of Internet Gaming Disorder, severity of problematic social media use, sleep quality and psychological distress. International Journal of Environmental Research and Public Health.

[B39-behavsci-15-01589] World Health Organization (2018). International classification of diseases for mortality and morbidity statistics.

[B40-behavsci-15-01589] Wu Y., Dai X. Y., Wen Z. L., Cui H. Q. (2010). Development of the adolescent learning burnout scale. Acta Psychologica Sinica.

[B41-behavsci-15-01589] Zhang A. (2021). The relationship between self-control and problem behaviors in elementary school students—The Mediating Role of Peer Relationships. Master’s thesis.

[B42-behavsci-15-01589] Zhang Y. L., Zhang Y. L., Zhang Y. X., Wang J. L., Huang C. Y. (2011). Reliability and validity of Chinese version of revised inventory of parent and peer attachment in junior students. Chinese Mental Health Journal.

